# Targeting neuroinflammation as a preventive and therapeutic approach for perioperative neurocognitive disorders

**DOI:** 10.1186/s12974-022-02656-y

**Published:** 2022-12-12

**Authors:** Chun Cheng, Hanxi Wan, Peilin Cong, Xinwei Huang, Tingmei Wu, Mengfan He, Qian Zhang, Lize Xiong, Li Tian

**Affiliations:** 1 Shanghai Key Laboratory of Anesthesiology and Brain Functional Modulation, Shanghai, 200434 China; 2 Translational Research Institute of Brain and Brain-Like Intelligence, Shanghai Fourth People’s Hospital, School of Medicine, Tongji University, Shanghai, 200434 China; 3 Clinical Research Center for Anesthesiology and Perioperative Medicine, Tongji University, Shanghai, 200434 China

**Keywords:** Perioperative neurocognitive disorders, Postoperative cognitive dysfunction, Neuroinflammation, Prevention, Treatment

## Abstract

Perioperative neurocognitive disorders (PND) is a common postoperative complication associated with regional or general anesthesia and surgery. Growing evidence in both patient and animal models of PND suggested that neuroinflammation plays a critical role in the development and progression of this problem, therefore, mounting efforts have been made to develop novel therapeutic approaches for PND by targeting specific factors or steps alongside the neuroinflammation. Multiple studies have shown that perioperative anti-neuroinflammatory strategies via administering pharmacologic agents or performing nonpharmacologic approaches exert benefits in the prevention and management of PND, although more clinical evidence is urgently needed to testify or confirm these results. Furthermore, long-term effects and outcomes with respect to cognitive functions and side effects are needed to be observed. In this review, we discuss recent preclinical and clinical studies published within a decade as potential preventive and therapeutic approaches targeting neuroinflammation for PND.

## Introduction

Perioperative neurocognitive disorders (PND) refers to a general term for cognitive impairment, which is identified during the perioperative period and often negatively affects multiple domains including memory, attention and concentration. PND is an umbrella term for the following conditions [1]: (i) neurocognitive disorders (NCD), a term of preoperatively diagnosed cognitive impairments; (ii) postoperative delirium, an acute event occurring in hours and days after surgery; (iii) delayed neurocognitive recovery and (iv) postoperative neurocognitive disorders, which are cognitive impairments diagnosed up to 30 days and 12 months after surgical procedures, respectively. The incidence of PND after noncardiac surgery reach to 41.4% at discharge and 12% at 3 months post-surgery in patients over 60 [2, 3], while it is higher after cardiac surgery especially with cardiopulmonary bypass (CPB), which is more than 50% and 24% at discharge and 6 months post-surgery, respectively [4, 5]. There is currently no standard preventive or therapeutic strategies for PND in clinical practice. However, growing evidence from both patients and animal models has indicated that neuroinflammation is a critical contributor to the pathogenesis and development of this problem, suggesting that neuroinflammation may be a target for developing novel therapies for PND. This review aims to comprehensively summarize and discuss the studies of anti-inflammatory approaches for PND or postoperative cognitive dysfunction (POCD) published within a period of 10 years, with a focus on potential mechanisms linking neuroinflammation and the problem, as well as the drug candidates aligning with these mechanisms.

### Search strategy and selection criteria

The literature search included terms “Perioperative neurocognitive disorder”, “Postoperative cognitive dysfunction” and “Anti-inflammatory”. Specifically for PubMed, the search strategy is ((Perioperative neurocognitive disorder) OR (Postoperative cognitive dysfunction)) AND (Anti-inflammatory). A total of 154 articles published in “English” between 2012 and 2022 were collected for subsequent screening. Additional articles were selected based on articles in these searches.

The search results were reviewed by two authors independently, and any discrepancies were evaluated by a third author. Duplicate studies from the same cohort were removed manually using endnote 20. Exclusion criteria were irrelevant topics, reviews, systematic review or meta-analysis, letters, case reports, commentaries and protocols.

### Neuroinflammation in the development of PND

Trauma of surgery and administration of anesthesia were well-documented to induce systemic inflammatory response [6], which subsequently influences the brain [7]. As two commonly used inhaled anesthetics, sevoflurane and isoflurane have been revealed to trigger and aggravate cognitive impairment with ample evidence from patients and animal models of PND. Sevoflurane-induced [8] and isoflurane-induced [9] neurotoxicity and neuroinflammation, which may be due to drug-induced proinflammatory cytokine release and microglial activation in the brain, have been previously revealed to participate in the development of PND, as demonstrated in aged rats. More specifically, sevoflurane-induced cognitive dysfunction was associated with downregulation of peroxisome proliferator-activated receptor ɣ (PPARɣ) [10], which could be retrieved by silencing of interferon regulatory factor 6 (Irf6) in hippocampal microglia [11]. Comparing to anesthetics, surgery may act as a more critical player to cognitive impairments, since it has been shown in a mouse model that laparotomy but not sevoflurane alone triggered peripheral and central inflammation as well as tau phosphorylation [12]. Extensive literature supports an important role of surgery-related inflammatory responses in the pathophysiology of POCD [13–15]. Meanwhile, surgical trauma causes dysfunction of endothelial cells and disruption of tight junctions (TJs), resulting in elevated permeability of the blood–brain barrier (BBB), which is also a critical player in the development of PND. The BBB is a multicellular vascular structure that protects the brain from the intrusion of toxins and pathogens, and its integrity is maintained by endothelial cell-formed continuous intracellular network of TJs. The disrupted BBB allows peripheral proinflammatory cytokines to be transmitted to the brain parenchyma to amplify the injury [16, 17]. Several mediators have been reported to contribute to the disruption of BBB integrity. Firstly, the activation of matrix metallopeptidases (MMPs) increases BBB permeability by down-regulating the expression of claudin-5 and occludin, which are two key components of TJs and critical determinants of BBB permeability [18]. Mice lacking MMP9 did not show surgical-induced negative effects that was observed in wild type mice [19]. Secondly, levels of hypoxia-inducible factor 1ɑ (HIF-1ɑ) and its target gene astrocyte-derived vascular endothelial growth factor (VEGF) were associated with BBB disruption and consequent cognitive impairment [20]. Of note, the amplification of neuroinflammation following surgically-triggered immune response is partially through the permeable BBB, while vagal afferent nerves and some other factors are also involved.

Neuroinflammation including microgliosis, astrogliosis and inflammatory cell ingress, particularly in hippocampus, has been proved to be main causes of PND [21–23], however, the evidence that neuroinflammation plays a role in human PND is much less clear than demonstrated in animal studies. First, under pathological conditions, microglia may be activated and play critical roles in neuroinflammation. Following anesthesia- and surgery-induced peripheral inflammation and BBB breakdown, microglia transform into hypertrophied cells to become “activated” microglia [24, 25], which can be classified into two phenotypes namely pro-inflammatory microglia (classically activated microphages) and anti-inflammatory microglia (alternatively activated macrophages) [26, 27]. The detrimental pro-inflammatory microglia have pro-inflammatory and phagocytic properties, secreting IL-6, IL-1β, inducible nitric oxide synthase (iNOS) and other mediators, while the protective anti-inflammatory phenotype have anti-inflammatory and tissue remodeling and repair properties, expressing IL-10, arginase 1 (Arg-1), Ym1, CD206, etc. [28–30]. Hence, activated microglia are a double-edged sword, and the pro-inflammatory/anti-inflammatory shifting is crucial for the modulation of neuroinflammation and adult neurogenesis in hippocampus. Treatment with commercially available recombinant human EPO (rhEPO) before and after abdominal surgery prevented POCD in mice by suppressing pro-inflammatory-related gene expression and promoting macrophage phenotype switching towards anti-inflammatory phenotype [31]. Furthermore, the activated microglia may promote the production of free radicals such as reactive oxygen species (ROS) and reactive nitrogen species (RNS), which contribute to oxidative stress in neurons and subsequent neurocognitive dysfunction [32, 33]. Second, astrocytes are also main contributors in neuroinflammatory response. Upon stimulation, astrocytes undergo morphological, transcriptional and functional changes to become reactive cells, which exhibit neurotoxic (A1) or neuroprotective (A2) properties. A1-astrocytes can be activated upon microglial activation and promotes neuronal death in neurodegenerative disorders [34] and pathogenesis at early stage of PND [35]. Therefore, when pro-inflammatory/anti-inflammatory microglia and A1/A2 astrocytes lose balance and the detrimental form becomes dominant, neuroinflammation is amplified and ultimately causes PND. Third, inflammatory cell ingress also promotes neuroinflammation. Monocyte chemoattractant protein 1 (MCP-1/CCL2) and its cognate receptor (CCR2) facilitate monocyte recruitment into tissues under infectious and sterile inflammatory conditions [36]. On one hand, CCR2-expressing macrophages were accumulated in hippocampi of mice undergoing experimental surgeries [37], and upregulation of CCL2 in activated astrocytes and elevated CCR2 expression in activated microglia induced cognitive deficits in a tibial-fracture-surgical model [38]. On the other hand, POCD manifestations are relieved in mice with attenuated CCL2 expression [39]. Therefore, targeting CCL2/CCR2 interaction may be a potential strategy to prevent PND.

Multiple signaling pathways, including high molecular group box 1 (HMGB1)/toll-like receptor (TLR) pathway, canonical nod-like receptor pyrin domain-containing 3 (NLRP3) inflammasome/caspase-1 pathway and non-canonical caspase 4/5/11 pathway, are involved in the pathogenesis of PND. The up-regulated protein level of HMGB1 was detected in hippocampus of rat brain following surgery and anesthesia [17]. HMGB1 acts as a damage-associated molecular pattern (DAMP) to bind to TLR and the receptor for advance glycosylation end product (RAGE) on circulating bone-marrow-derived monocytes (BM-DM) and endothelial cells, triggering the production of pro-inflammatory cytokines and facilitating leukocyte migration and immune cell recruitment, resulting in neuroinflammation and PND [40, 41]. Therefore, neutralizing antibodies to HMGB1 may be a potential treatment for PND. The NLRP3 inflammasome/caspase-1 and non-canonical caspase 4/5/11 pathways have been reported to be involved in the pathogenesis of PND by triggering pyroptosis, a recently characterized inflammatory form of programmed cell death. Furthermore, NLRP3 inflammasome activity was negatively regulated by autophagy [42, 43]. Therefore, targeting NLRP3 inflammasome could be a new preventive and therapeutic strategy for PND.

### Drug candidates targeting systemic inflammation/inflammation amplification

**Fig. 1:**
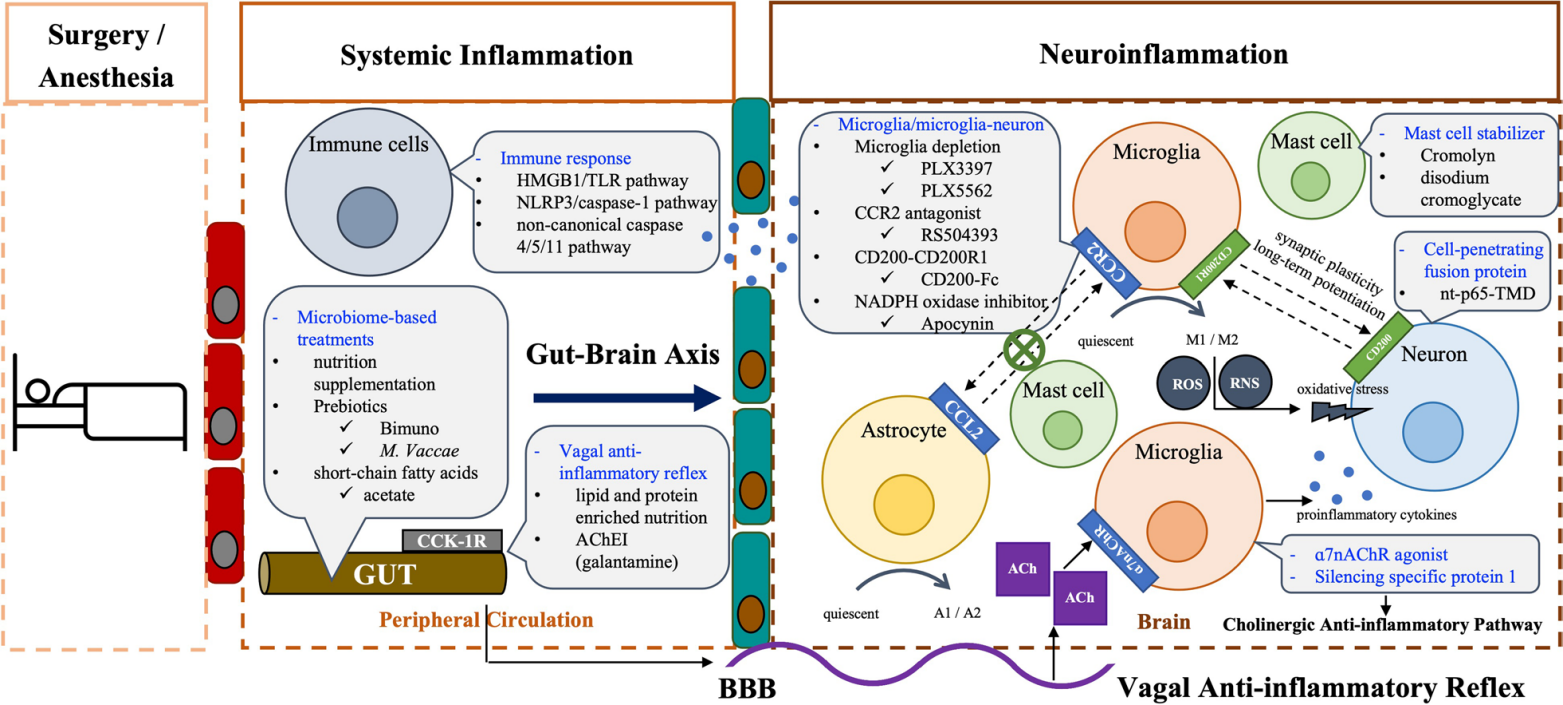
From systemic inflammation towards neuroinflammation. Trauma of surgery and administration of anesthesia induced systematic response, resulting in an increased production and release of proinflammatory mediators (dots in blue). Surgeries induced accumulation of CCR2-expressing macrophages in hippocampi, then upregulated CCL2 in activated astrocytes and CCR2 in activated microglia, which could be abolished by CCR2 antagonist. The vagus nerve was conducted to release ACh, which binds to ɑ7nAChR and reduce the production of proinflammatory cytokines. The vagal anti-inflammatory reflex could be activated by enteral administration of boluses of lipid and protein enriched nutrition, which could stimulate CCK-1R in the gut to activate the vagal afferent nerves to inhibit proinflammatory cytokine release. Meanwhile, silencing specific protein 1 or ɑ7nAChR agonists exert beneficial effects via reactivating cholinergic anti-inflammatory pathway. The brain mast cell stabilizers are capable of attenuating POCD via inhibiting astrocyte activation and microglia-astrocyte communication. Enteral administration of lipid and protein enriched nutrition has been reported to prevent PND via vagal anti-inflammatory reflex, besides, microbiome-based treatments including nutrition supplementation, prebiotics and SCFAs exert modulatory effects through the Gut-Brain Axis

Systemic inflammation may exert relatively long-term effects on the brain, and multiple animal studies indicated that anti-inflammatory treatment could attenuate POCD development [44, 45]. However, these studies have not yielded any clinically effective treatment. This may be explained by the results obtained in a bile duct ligation model of POCD [14], which suggested that inhibition of peripheral inflammation would be insufficient to recover cognitive impairment [46]. Once microglial activation achieved, they may play a dominant part in sustaining neuroimmune responses and resulting in neurocognitive impairments (Fig. 1).

### Candidates on signaling pathways regulating immune response

#### NLRP3

Injection of Ac-YVAD-cmk (an NLRP3/caspase 1 inhibitor) prior to anesthesia did improve cognitive impairments and prevent hippocampal inflammation in aged mice, but not in young mice, which was possibly due to the attenuation of isoflurane-triggered NLRP3 inflammasome [9]. Moreover, Elamipretide (SS-31) (a mitochondrial-targeted peptide) has shown protective effects against post-surgery cognitive deficits in aged mice subjected to laparotomy, which involves multiple mechanisms including rescuing surgery-induced mitochondrial dysfunction, NLRP3/caspase 1-dependent pyroptosis, neuronal damage and downregulation of synaptic integrity in hippocampus [47].

#### Toll-like receptors and HMGB1

A recent study showed that rats undergoing cardiac surgery with CPB could be protected against neurological damage in spatial learning and memory abilities and brain damage in hippocampus by antler MSCs (AMSCs)-derived exosomes via inhibiting TLR2/TLR4 signaling pathway and preventing inflammatory response, oxidative stress and neuronal apoptosis [48]. When mice subjected to right carotid artery exposure under isoflurane anesthesia were treated with a cell permeable TLR1/TLR2 dual antagonist CU-CPT22, or with a natural triterpene glycoside and a HMGB1 antagonist glycyrrhizin, both drugs attenuated TLR2-contributed neuroinflammation and subsequent dysfunction of hippocampus-dependent spatial learning and memory following surgery [49]. In addition, selected TLR4 inhibitor TAK-242 has been shown to reverse decline in freezing behavior as well as elevation of TNF-ɑ and IL-1β protein expression post-operatively in db/db mice that underwent tibial fracture surgery [50].

### Microbial-based treatments

#### Nutrition-based therapies

After enteral supplementation of fat/protein-enriched nutrition to surgery-induced POCD rat models, inhibition of systematic inflammation and improved long-term spatial learning and memory was only observed in young rats, but not in old rats, while a reduction of neuroinflammation was absent in both age groups [51]. In addition, treatment with polydeoxyribonucleotide (PDRN) extracted from salmon sperm on human neuronal SH-SY5Y cells in POCD conditions activated adenosine A2A receptors and promoted the phosphorylation of cAMP response element-binding protein (CREB) through the cAMP-dependent protein kinase A (PKA) pathway, then significantly reduced proinflammatory cytokines (TNF-ɑ, IL-1β, IL-6) and increased the expression of VEGF and brain-derived neurotrophic factor (BDNF), which was reduced by lipopolysaccharide (LPS) and sevoflurane exposure [52].

### Prebiotics

Prebiotics, which are defined as a collection of substrates that can be selectively utilized by host microorganisms to manipulate gastrointestinal microbiota to regulate host immunity as well as cognition via gut-brain axis [53]. The prebiotic Bimuno (galactooligosaccharide (B-GOS) mixture) is a widely investigated specific nondigestible mixture particularly designed for selective promotion of the proliferation of *Bifidobacterium* [54]. In adult rats undergoing abdominal surgery under isoflurane anesthesia, Bimuno significantly alleviated cognitive decline and downregulated microglial activation, which is associated with a dramatic change of β-diversity of gut microbiome and proliferation of *Bifidobacterium* and other potentially anti-inflammatory microbes [55]. In addition to this mixture, prebiotics with specific microbes have also been tested. For instance, *Mycobacterium vaccae* (*M. vaccae*), a fast-growing and widely distributed species of saprophytic bacteria found in soil, can modify immune response in both humans and rodents. Immunizing adult rats with a heat-killed preparation of *M. vaccae* protected against stress-elicited, primed, hyperactive immune responses and accompanying stress-induced behavioral impairments [56, 57]. Heat-killed *M. vaccae* (NCTC11659) immunization prior to surgery shifted the pro-inflammatory hippocampal microenvironment towards an anti-inflammatory phenotype, consequently prevented post-operative learning/memory deficits in a fear conditioning paradigm in aged (but not young) rats, possibly through upregulating IL-4 signaling [58].

### Short-chain fatty acids

Acetate, one of the short-chain fatty acids (SCFAs), has been reported to exert antioxidant activity by reducing LPS-induced nitric oxide production in rat primary astrocytes [59], and inhibit inflammatory responses in different models [60, 61]. In both in vivo and in vitro PND models, acetate treatment successfully exhibited beneficial effects against PND by suppressing microglial activity through binding to GPR43, and simultaneously reducing expression of inflammatory proteins, oxidative stress factors and signaling molecules in hippocampus [62].

### Targeting vagal anti-inflammatory reflex

Surface receptor ɑ7 nicotinic acetylcholine receptor (ɑ7nAChR) is widely distributed in the central and peripheral nervous systems, especially in prefrontal lobe, ventral tegmental area and hippocampus, to regulate cognition, learning, memory, emotional behavior, etc. The vagus nerve was conducted to release acetylcholine (ACh), which binds to ɑ7nAChR and reduces the production of pro-inflammatory cytokines by inhibiting NF-κB activity [63, 64]. A previous study demonstrated that activation of ɑ7nAChR could improve POCD via vagal anti-inflammatory reflex [65]. It was also reported that the vagal anti-inflammatory reflex could be activated by enteral administration of boluses of lipid and protein enriched nutrition [66, 67]. These nutrients were capable of stimulating cholecystokinin (CCK)-mediated CCK-1 receptor in the gut to activate the vagal afferent nerves and subsequently inhibit proinflammatory cytokine release via nicotinergic acetylcholine receptors [67, 68]. Such an inhibition of proinflammatory cytokines and promotion of anti-inflammatory cytokines has also been observed in humans after postpyloric administration of nutrition enriched with lipid and specific proteins [69]. Silencing specificity protein 1 (SP1) is another way to alleviate sevoflurane-induced POCD in both in vivo and in vitro models via rescuing the deactivation of cholinergic anti-inflammatory pathway (CAP) [70]. The use of ɑ7nAChR agonist led to an increased release of anti-inflammatory mediators and a reduced pathological damage in peripheral and brain tissues [71], suggesting that the neuroprotective mechanism of CAP may be dependent on ɑ7nAChR. Furthermore, as an AChEI, galantamine upregulates extracellular levels of ACh by inhibiting hydrolysis of ACh, thereafter counteracts deficiency of cholinergic innervation. When performing stabilized tibial fracture operation in male mice, daily intraperitoneally administration of galantamine did alleviate microglial accumulation in hippocampus and normalize excitatory synaptic transmission to exert beneficial effects on reversing cognitive dysfunction in a fear conditioning paradigm in these mice [72].

### Drug candidates targeting neuroinflammation

Neuroinflammation triggers the development of PND, which could be targeted to develop preventive and therapeutic strategies for PND (Fig. 1). However, research in this area is still at pre-clinical stage (Table 1).

**Table 1:** Drugs targeting microglia and neuroinflammation amplification

Drug type	Drug name	Study subject/surgery (anesthetic)	Functional outcome	Potential mechanisms	Refs.
Microglia depletion	PLX3397	Mice/etomidate-induced	Rescued cognitive impairment	Suppressed A1-specific astrocytic response during the early but not the late stage of PND	[35]
	PLX5622	Mice/TF (ISO)	Did not affect learning and memory	Reduced hippocampal levels of inflammatory cytokines, abrogated microglial activation and hippocampal recruitment of CCR2 leukocytes	[73]
CCR2 antagonist	RS504393	Rats/TF (ISO)	Abrogated surgery-induced cognitive deficits	Abolished M1 microglial polarization and subsequent neuronal loss	[38]
CD200 fusion protein	CD200-Fc	PND model	Attenuated neuroinflammation and PND	Improved synaptic plasticity and long-term potentiation	[76]
NADPH oxidase inhibitor	Apocynin (APO)	Mice/EL (ISO)	Alleviated impaired contextual fear memory	Rescued surgery-associated brain pathology (including increased Nox2, 8-OH-dG, CD11b, IL-1β and decreased BDNF in hippocampus)	[80]
Nutrition	lipid and protein enriched nutrition, enteral administration	Mice		Stimulated CCK-1R in the gut, inhibited proinflammatory cytokine release	[67, 68]
Specific protein 1	SP1 knockdown	Rats/no surgery (SF)		Attenuated sevoflurane-induced SP1 up-regulation, reactivated CAP	[70]
mast cell stabilizer	Cromolyn (disodium cromoglycate)	Rats/TF	Attenuated surgery-induced cognitive impairments	Attenuated surgery-induced astrocyte activation and microglia-astrocyte communication, inhibited BBB permeability increase, alleviated reduction of occluding and claudin-5 levels, neutralize hippocampal expression of MMP-2 and MMP-9	[83]
Cell-penetrating fusion protein	nt-p65-TMD	Mice	Attenuated surgery-induced cognitive decline	reduced surgery-induced elevations in cerebrovascular integrity impairment, subsequent peripheral immune-cell recruitment, and inflammation amplification, regulated and reduce systemic inflammation and inflammation amplification	[85]

*EL* exploratory laparotomy, *TF* tibial fracture, *ISO* isoflurane anesthesia, *SF* sevoflurane

### Depletion of microglia and microglia-neuron interactions

#### Microglia depletion

The depletion of microglia in the central nervous system (CNS) was fulfilled by inhibitors of colony-stimulating factor 1 receptor (CSF1R), including PLX3397 and PLX5622. In PND mouse models, PLX3397 successfully reduced A1-specific astrocytic response and rescued cognitive impairment at early but not late pathological stage [35], whereas PLX5622 remarkably protected mice undergoing tibial fracture from POCD by reducing hippocampal levels of inflammatory cytokines, and abrogating microglial activation and hippocampal recruitment of CCR2 leukocytes [73], which are often accumulated after surgical challenge [37]. Therefore, targeting microglia before surgery might be effective to prevent PND in vulnerable or elderly patients. However, the impact of CNS microglia suppression on humans requires to be further carefully investigated.

#### CCR2 antagonist

Apart from microglial depletion, disruption of CCL2/CCR2 interaction might be another strategy of choice. When performing site-directed pre-injection on rats with RS504393, a CCR2 antagonist, the drug abrogated surgery-induced cognitive deficits and abolished pro-inflammatory microglial polarization and subsequent neuronal loss [38].

#### CD200-CD200R1

The interaction between CD200 (a neuronal surface protein) and CD200R1 (receptor of CD200 on microglia) is important for the maintenance of the quiescent state of microglia. CD200 deficiency may lead to pro-inflammatory microglia activation [74], neuroinflammation and synaptic dysfunction [75], whereas CD200R1 activation either by its agonist or IL-4 may result in the promotion of anti-inflammatory phenotype in innate immune cells including macrophages and microglia to resolve an inflammatory response [76, 77]. Apart from ageing, decreased CD200 mRNA level was also detected in a PND model [78]. Therefore, researchers have attempted to inject a CD200 fusion protein (CD200-Fc) into the lateral ventricle of the model, which attenuated neuroinflammation and PND with improved synaptic plasticity and long-term potentiation (LTP) [79].

#### NADPH oxidase inhibitor

Neurons are susceptible to ROS, which are mainly derived from NADPH oxidase 2 (NOX2). Since oxidative stress plays a critical part in neuronal dysfunction in the development of PND, the NADPH oxidase inhibitor apocynin (APO) has been tested in mice subjected to exploratory laparotomy with isoflurane anesthesia, and the drug alleviated surgery-induced impaired contextual fear memory as well as associated brain pathology [80].

### Brain mast cell stabilizer

Brain mast cells are located perivascularly in proximity to neurons and microglia in the CNS and are the first responder to injury. Despite their small numbers, the activation of mast cells following an cerebral ischemic event had a dramatic effect on BBB breakdown [81], whereas a tibial fracture surgery may induce brain mast cell degranulation, microglial activation and neuroinflammation [82]. Based on these investigations, the brain mast cell stabilizers cromolyn (also disodium cromoglycate) was injected into rats undergoing open tibial fracture surgery, showing attenuation of surgery-induced cognitive impairments as well as astrocyte activation and microglia-astrocyte communication [83]. Additionally, the inhibition of surgery-triggered increase of BBB permeability, the alleviation of surgery-induced reduction in occludin and claudin-5 levels within hippocampus, and the neutralization of hippocampal expression of MMP-2 and MMP-9 have also been observed after cromolyn treatment on surgery-exposed rats [84].

### Cell-penetrating fusion protein

Protein transduction domains (PTD), which can translocate into cells and allow them to transport other large molecules into the cells, are also used as a potential treatment for POCD. For instance, cell-penetrating fusion protein called nt-p65-TMD is a novel chemical-conjugated form of NF-κB subunit p65 that contains cell-permeable peptides. A previous study has revealed that NF-κB attached Hph-1-PTD (nt-p65-TMD) could easily be delivered into cells and tissues, allowing it to directly target endogenous p65 in an interatomic inhibitory manner without inducing cytotoxicity [85]. The nt-p65-TMD has also been tested on a POCD mouse model, resulting in a reduction of surgery-induced impairment of cerebrovascular integrity and amplification of systemic inflammation [85].

### Perioperative drugs for prevention and treatment of PND

Perioperative medications including analgesics, muscle relaxant antagonists, antibiotics, as well as some non-pharmacological techniques are also reported as potential preventive and therapeutic approaches for PND (Table 2).

**Table 2:** Perioperative drugs for prevention and treatment of PND

Drug type	Drug name (dose)	Study subject/surgery (anesthetic)	Functional outcome	Potential mechanisms	Refs.
Animal studies					
Acetaminophen	Acetaminophen (APAP)	Mice/LPS	Ameliorated LPS-induced cognitive impairment	Suppressed proinflammatory cytokine accumulation and microglial activation, increased SOD activity, reduced MDA, modulated GSK3β activity, elevated BDNF, decreased Bax/Bcl-2 ratio and neuron apoptosis in hippocampus	[86]
NSAIDS	Ibuprofen	Aged mice/abdominal surgery (local anesthesia)	Ameliorated surgery-induced cognitive impairment in aged mice	Attenuated surgery-induced levels of TNF-ɑ, IL-6, Iba1 positive cells in hippocampus	[87]
	Ibuprofen (60 mg/kg) preoperative single injection	Mice/sevoflurane alone or laparotomy (sevoflurane)	Improved cognitive performance	attenuated systemic inflammation and glial activation, suppressed abnormal tau phosphorylation both in the frontal cortex and hippocampus	[12]
	Ibuprofen (15 mg/kg) preoperative single injection	Young and aged mice/abdominal surgery	Improved short-term but not long-term spatial memory, less pronounced effects in aged mice	Increased hippocampal neurogenesis, but neuroinflammation and gut microbiome was not affected	[88]
	Ketoprofen postoperative vs. morphine	Mice/laparotomy (ISO)	Similar pain-relieving effects and attenuation of memory dysfunction		[89]
Alpha-2 agonist	DEX 20 μg/kg, 20 min before surgery	Aged mice/EL (ISO)	Alleviated surgery-induced cognitive impairment on POD3 and POD7	Suppressed proinflammatory cytokine accumulation and microglial activation, reduced MDA, enhanced SOD activity, modulated CDK5 activity, increased BDNF, decreased Bax/Bcl-2 ratio and apoptotic neurons in hippocampus	[94]
	DEX 10,20,30 μg/kg, once a day for 3 days before surgery	Aged mice/EL (ISO)	Improved surgery-induced learning and memory, relieved decrease in spine density	Reduced IL-1β secretion, promoted autophagic process of microglia, reduced NLRP3-mediated inflammation by accelerating its ubiquitination and degradation	[95]
	DEX 20 μg/kg, 30 min before surgery	Aged mice/SP (pentobarbital)	Attenuated cognitive deficits	Reduced TNF-ɑ and IL-1β, DEX-regulated lncRNA LOC102546895 may play a key role in POCD, promoted apoptosis in microglial cells, promoted target gene (Npas4) expression	[96]
	DEX 3,12 μg/kg, after anesthesia	Aged mice/EL (ISO)	Rescued cognitive impairment	Rescued inflammatory changes, increased expression of BDNF, PKA, p-CREB/CREB	[97]
COX-2 inhibitor	Parecoxib	Rats	Improved cognitive function	Inhibited COX-2 overexpression	[103]
	Meloxicam 60 mg/kg, 24 h after surgery	Mice/SP	Attenuated surgical-reduced object recognition at POD5 and POD9	Reduced microglial activation	[108]
Gabapentinoids	Pregabalin	Aged rats/abdominal surgery	Reduced post-surgery memory deficits	Reduced neuroinflammation	[123]
AChEI	physostigmine vs. neostigmine	Rats/surgery and LPS		Decreased IL-1 and TNF-ɑ, reduced surgery- and LPS-induced neurodegeneration and increased activity of AChE in the cortex and hippocampus	[129]
Antibiotic	Minocycline preoperative treatment	Aged rats/TF (ISO)	Attenuated surgical-induced memory dysfunction with fear conditioning	Suppressed microglial overactivation and release of hippocampal pro-inflammatory cytokines	[132]
	Minocycline postoperative treatment for 30 days	Rats/CPB	Improved performance in behavioral testing (win-shift task on an 8-arm radial maze) at 6 months post CPB	Inhibited hippocampal microglial activation, attenuated surgery-reduced neurogenesis	[133]
	Cefazolin	Mice/EL	Attenuated surgery-induced cognitive impairment in memory and learning	A direct anti-inflammatory effect	[134]
Non-pharmacologic	Electroacupuncture to the Baihui acupoint (GV20) twice daily for 7 days after surgery	Aged mice/partial hepatectomy surgery	Alleviated POCD-mediated cognitive dysfunction	Alleviated neuroinflammation, inhibited NLRP3	[147]
Clinical trials					
Alpha-2 agonist	DEX	Adult pts with stable femoral neck fractures/Internal fixation surgery	Higher quality of recovery scores, satisfaction with pain management and MMSE scores; lower disability scores and pain anxiety; less catastrophic thinking, opioid intake, emergence agitation and incidence of POCD, measured on POD1 and POD2		[98]
	DEX (0.1 μg/kg/h) from ICU admission till 0800 on POD1	Elderly pts/elective noncardiac surgery	Reduced delirium incidence during the first 7 days after surgery		[99]
	DEX (0.3 μg/kg/h) intraoperative infusion	Elderly pts/radical resection of colorectal cancer	Reduced POCD incidence and degree of cognitive dysfunction		[100]
	DEX (0.5 μg/kg/h) during and for 2 h in recovery room	Elderly pts/major elective noncardiac surgery	Did not reduce delirium incidence or improve cognitive performance at 3- and 6-month follow-up		[101]
	DEX (0.4 μg/kg/h) intraoperative infusion and (0.1 μg/kg/h) postoperative infusion	Elderly pts/elective cardiac surgery	Did not reduce delirium incidence during the first 5 days after surgery		[102]
COX-2 inhibitor	Parecoxib sodium 40 mg/time, shortly after induction and 12 h post-surgery	Elderly pts/total knee arthroplasty	Significantly reduced POCD incidence	Reduced plasma IL-1β, IL-6, TNF-ɑ, no difference in plasma CRP	[104]
	Parecoxib + DEX	Pts/laparoscopic cholecystectomy surgery	Combined treatment reduced postoperative pain and improved postoperative sedation and cognition conditions		[105]
	Parecoxib (40 mg) + DEX (0.5 μg/kg/h)	Pts/shoulder arthroscopy	Combined treatment reduced early POCD incidence, improved postoperative analgesia effect and cerebral oxygen metabolism		[106]
	Celecoxib	Elderly pts/total knee arthroplasty	Reduced POCD incidence on POD7 but not at 3 months, reduced acute postoperative pain	Reduced plasma COX-2, IL-1β, IL-6, TNF-ɑ, S100β	[107]
NMDA antagonist	ketamine (0.5 mg/kg) during anesthetic induction vs. placebo	Elderly pts/cardiac surgery with CPB	Reduced delirium incidence	Reduced postoperative serum CRP	[111]
	ketamine (1 mg/kg/h) infusion vs. propofol (1.5-6 mg/kg/h) infusion	Pts/cardiac surgery with CPB	Lower delirium incidence in ketamine group		[114]
	ketamine low dose (0.5 mg/kg), high dose (1.0 mg/kg) after induction vs. placebo	Elderly pts/major cardiac and non-cardiac surgery	Did not reduce delirium incidence in the first 3 days after surgery		[113]
Glucocorticoid	Dexamethasone (0.1 mg/kg) 10 h before surgery	Pts/elective cardiac surgery	Reduced POCD incidence both in the short-term (on POD6) and in the long-term (4 years)		[115, 116]
	Dexamethasone (8 mg) prophylactic intravenous bolus	Pts/non-cardiac non-neurologic surgery	Reduced POCD incidence when administered with bispectral index (BIS) 46–55, but not 35–45		[117]
	Dexamethasone (8 mg) before induction, (8 mg) every 8 h for 2 days	Pts/cardiac surgery	Reduced delirium risk		[118]
	Dexamethasone (0.2 mg/kg) preoperative intravenous bolus	Pts with facial spasm/microvascular decompression	Increased POCD incidence on POD5		[119]
	Dexamethasone (1 mg/kg) intraoperative intravenous bolus	Pts/cardiac surgery with CPB	Did not reduce POCD incidence at 1 month or 12 months after surgery		[120]
	Methylprednisolone (250 mg) intraoperative administration at induction and before CPB	Pts/cardiac surgery with CPB	Did not reduce delirium incidence or improve QoR		[121]
AChEI	Neostigmine (0.04 mg/kg) when TOFR ≤ 0.5, (0.02 mg/kg) when TOFR > 0.5	Elderly pts/radical section of GI cancer	Reduced early POCD incidence	Did not affect peripheral inflammatory factors (IL-1β, IL-6, TNF-ɑ)	[124]
	Sugammadex vs. neostigmine/atropine	Adult pts/elective surgery	Similar POCD incidence at 1 h post-surgery and at discharge		[128]
	Sugammadex vs. neostigmine	Pts/cardiac surgery with enhanced recovery after cardiac surgery	Sugammadex improved postoperative cognition	Data from animal model showed sugammadex induced expression of anti-inflammatory markers	[127]

*EL* exploratory laparotomy, *TF* tibial fracture, *SP* splenectomy, *ISO* isoflurane anesthesia, *POD* postoperative day, *DEX* Dexmedetomidine, *pts* patients

### Analgesics

#### Acetaminophen

As a widely used analgesic and antipyretic, acetaminophen (N-acetyl-4-aminophenol, also known as APAP or paracetamol) has been demonstrated in mice to exert antioxidant, anti-inflammatory and neuroprotective effects and to improve LPS-induced cognitive impairment by inhibiting mitochondrial permeability transition (MPT) pore and subsequent apoptotic pathway [86].

#### NSAIDS

Ibuprofen is one the of most commonly-used non-steroid anti-inflammatory drugs (NSAIDs) and has been tested for its indication to treat POCD in different animal models. Interestingly, ibuprofen has been shown to ameliorate peripheral-surgical-wounding-induced cognitive impairment in aged mice subjected to abdominal surgery under local anesthesia without the influence of general anesthesia, in which age-dependent neuroinflammation and β-amyloid accumulation have been induced [87]. Under general anesthesia with sevoflurane, preoperatively administered ibuprofen to mice has improved postoperative cognitive performance in association with a long-lasting inhibition of both systemic- and neuro-inflammation, as well as suppression of abnormal tau phosphorylation in frontal cortex and hippocampus [12]. However, in another study, a single injection of ibuprofen only improved short-term but not long-term spatial memory after surgery, with neurogenesis increased but without affecting neuroinflammation or gut microbiome, and less pronounced in aged rats [88]. Ketoprofen has also been tested on mice undergoing surgery performed under isoflurane in comparison with morphine. The postoperative analgesia of ketoprofen has been shown to prevent the development of surgery-associated memory deficits to a similar degree as that of morphine via its pain-relieving effects [89].

#### Alpha-2 agonists

Dexmedetomidine (DEX) is a highly selective alpha-2 adrenergic receptor agonist with dose-dependent hypnotic, sedative, antiemetic and analgesic effects [90]. DEX facilitates acetylcholine secretion by binding to ɑ7nAChR, thereby inhibiting inflammatory cytokine release [91, 92] and mitigating excessive neuronal inflammatory responses to prevent the development of POCD [93]. Apart from its anti-inflammatory property, mounting evidence (see Table 2) from animal studies have also demonstrated the neuroprotective effects of DEX against surgery-induced cognitive impairment through mechanisms involving antioxidant, anti-apoptotic and inhibitory effects on MPT pore [94–97]. Meanwhile, this drug also promoted autophagic process of microglia and reduced NLRP3-mediated inflammation by accelerating its ubiquitination and degradation [95]. In addition, DEX also plays a proneurogenesis role in the prevention of POCD by upregulating the expression of BDNF, PKA, p-CREB/CREB and following p-P38-MAPK [97]. The whole transcriptome sequencing reveals that DEX-regulated long non-coding RNA (lncRNA) LOC102546895 may contribute to the development of POCD by targeting Npas4 and promoting apoptosis of microglial cells [96]. The effects of DEX against PND have been studied in randomized controlled trials (RCT), which have yielded inconsistent results. For instance, measured on the first and second day after internal fixation surgery on patients with stable femoral neck fractures, DEX as an adjunct to anesthesia significantly lowered incidence of POCD as well as pain-related anxiety and agitation, and obviously improved overall satisfaction with pain management and quality of recovery (QoR), suggesting that DEX may change the post-surgery pain management strategy towards improved cognitive dysfunction [98]. Similarly, in another trial of elderly patients undergoing elective noncardiac surgery, prophylactic lower dose of DEX (0.1 μg/kg/h) significantly reduced incidence of delirium within the first week after surgery [99]. In addition, intraoperatively maintaining DEX also significantly lowered POCD incidence among elderly patients undergoing radical resection of colorectal cancer [100]. However, negative results have been obtained as well. In another trial with elderly patients undergoing major elective noncardiac or cardiac surgery, the intraoperative DEX infusion at higher dose (0.4–0.5 μg/kg/h) did not significantly alter the incidence of delirium within 5 days post-surgery or cognitive performance at 3- and 6-month-follow-up [101, 102].

#### COX-2 inhibitors

Parecoxib, a highly selective cyclooxygenase (COX-2) inhibitor, has been demonstrated to improve cognitive function in POCD rats via inhibiting COX-2 overexpression [103]. A significantly lowered POCD incidence was obtained when treating elderly patients undergoing total knee arthroplasty with the drug shortly after induction of general anesthesia and 12 h after the surgery [104]. Apart from a decrease of early POCD incidence, additional benefits including reduced postoperative pain and improved postoperative sedation have been observed when administered with DEX on patients after laparoscopic cholecystectomy [105] or scheduled shoulder arthroscopy [106]. Celecoxib is another highly selective COX-2 inhibitor that provides anti-inflammatory and analgesic effects both in a COX-2-dependent and -independent manner. For geriatric patients undergoing total knee arthroplasty, celecoxib treatment decreased an early POCD incidence (on postoperative day (POD)7) and an acute postoperative pain, in association with reduced plasma levels of COX-2 and proinflammatory markers, but no such benefit was detected at 3-month follow-up any further [107]. Another selective COX-2 inhibitor, meloxicam, was found to be effective in the treatment of surgery-mediated neuroinflammation and cognitive decline (such as object recognition memory) in animal studies, which may depend on the modulation of glial cell activation [108].

#### NMDA antagonists

Ketamine has shown potential to reduce the incidence of surgery-induced delirium in human and animal studies, based on its strong anti-inflammatory properties [109–111], neuroprotective actions [112], and rapid and lasting anti-depressant actions [113]. When added to routine anesthetics in patients undergoing cardiac surgery, ketamine significantly lowered incidence of delirium in comparison with placebo [111] or propofol [114]. However, a large-scaled study in both cardiac and non-cardiac surgery using only single dose of ketamine after induction of anesthesia did not affect the outcome in the first 3 days after surgery [113].

#### Glucocorticoids

Dexamethasone is a potent synthetic glucocorticoid with a long duration of action and a biological half-life of 36–54 h. Dexamethasone is commonly used in perioperative settings, owning to its antiemetic properties and its ability to reduce airway swelling and fatigue. Its beneficial effects on POCD prevention have been shown in clinical studies. When prophylactically administering a single intravenous bolus of 0.1 mg/kg dexamethasone to patients before elective cardiac surgery, a lower incidence of POCD both in the short-term (on POD6) [115] and in the long-term (4 years after surgery) [116] was observed by reducing inflammatory responses. When administered with bispectral index (BIS) 46–55, but not 35–45, a single intravenous bolus of 8 mg prophylactic dexamethasone could help to preserve most cognitive functions (especially memory and executive function) and reduce POCD incidence after noncardiac non-neurologic surgery [117]. Administration of repeated intravenous doses (8 mg before induction and 8 mg every 8 h for 2 days) to patients undergoing cardiac surgery significantly reduced the risk of delirium [118]. However, contradictory results have been observed for higher doses. Preoperative administration of 0.2 mg/kg dexamethasone on patients with facial spasm undergoing microvascular decompression (MVD) showed a higher incidence of POCD on POD5 [119], while intraoperative administration of 1 mg/kg dexamethasone on patients undergoing cardiac surgery with CPB demonstrated no beneficial effects on POCD incidence at either 1 month or 12 months after surgery [120]. Methylprednisolone is another glucocorticoid tested in the conditions of POCD. A high-dose (250 mg) intraoperative administration during induction and before CPB respectively neither reduced delirium nor improved QoR in high-risk cardiac surgical patients [121].

#### Gabapentinoids

Pregabalin was initially developed as an anticonvulsant for epilepsy, and later for neuropathic pain [122]. Pregabalin treatment during the early postoperative period on aged rats undergoing abdominal surgery could prevent neuroinflammation and post-surgery memory deficits, possibly through an interaction between peripheral and central neuroimmune systems, but not via direct anti-inflammatory effects [123].

### Muscle relaxant antagonists

Acetylcholinesterase inhibitors (AChEIs) prevented acetylcholinesterase (ACh) from breaking down acetylcholine, thus enhancing cholinergic transmission. As a commonly used muscle relaxant antagonist, Neostigmine significantly reduced the risk of POCD without affecting peripheral inflammatory factors [124]. Of note, neostigmine was commonly administered in combination with anticholinergic agents, such as glycopyrrolate and atropine, to reverse neuromuscular blockade. Considering that these agents are able to pass the BBB and may lead to disturbances in the cholinergic transmission and subsequent central cholinergic deficits [125], these agents used in combination may contradict the potential beneficial effect of neostigmine. However, the long-term side effects of neostigmine remain unknown, and it is also not clear whether neostigmine directly reduced neuroinflammation in the CNS, since it does not cross the BBB. Sugammadex (SG) is a modified ɣ-cyclodextrin designed for optimal encapsulation of the neuromuscular blockade agent blocking drug rocuronium [126], which is associated with faster recovery of consciousness after general anesthesia [127]. SG could not cross the BBB either due to its high molecular weight. In one RCT, SG as a neuromuscular block reversal was shown to have a similar POCD incidence to neostigmine/atropine treatment [128]. When comparing to neostigmine in another RCT, SG demonstrated a favorable recovery in cognition domains particularly at POD30 in patients undergoing cardiac surgery with enhanced recovery after cardiac surgery approach, and the effect over glial cells was proposed as the underlying mechanism [127]. Apart from neostigmine and SG, which act only peripheral, another AChEI physostigmine, which crosses the BBB, showed significant alleviation of surgery- and LPS-induced pro-inflammatory responses and neurodegeneration in cortex and hippocampus in rats [129].

### Antibiotics

Minocycline, a second-generation tetracycline derivative which can cross the BBB, has been reported to have neuroprotective effects via inhibiting inflammation and differentiation of pro-inflammatory but not anti-inflammatory microglia [130, 131]. When treating aged mice with minocycline prior to surgery of the tibia, it was capable of attenuating isoflurane- and surgery-induced cognitive impairment in spatial learning memory by suppressing microglial overactivation and release of hippocampal pro-inflammatory cytokines, indicating that minocycline may be an effective and practical intervention for POCD prevention [132]. In a recent study, daily administration of minocycline to rats for 30 days post CPB resulted in a significantly better performance in behavioral testing at 6 months after surgery, which was in association with a reduced number of activated microglia/macrophages in hippocampus and a prevention of CPB-induced reduction in adult neurogenesis [133]. Cefazolin is often used for prevention of perioperative infection. This antibiotic has been tested on mice subjected to laparotomy, showing a direct anti-inflammatory effect and attenuation of surgery-induced impairment in memory and learning [134]. However, we should be cautious about the use of cefazolin since it has been reported to induce cognitive dysfunction possibly by transient gut dysbiosis in mice without surgery [134].

### Comparing anesthesia types

When comparing general and regional anesthesia on patients undergoing total knee arthroplasty (TKA), regional anesthesia yielded better performance in neurocognitive tests compared to general anesthesia, which may be associated with lower cortisol and glucose levels and higher insulin levels [135]. In animal models, inhalational anesthetic drugs are reported to cause deficits in learning and memory by promoting neuronal apoptosis [136]. In elderly patients undergoing major surgery, as compared to those maintained on intravenous propofol, POCD incidence was higher in those under inhalational anesthesia with sevoflurane and lower in those pro-treated with methylprednisolone before sevoflurane anesthesia [137]. Moreover, the elevated POCD incidence in those receiving sevoflurane anesthesia was indicated by elevated plasma concentrations of S-100β protein, TNF-ɑ and IL-6 [137]. The superior effect of intravenous propofol in post-exposure cognitive function is also reported in patients undergoing minor surgeries [138]. Notably, the results from recent studies have suggested that the choice of type of anesthesia dose not influence clinical outcomes of cognition. For instance, none of the regional anesthesia (spinal, epidural or both) was superior to general anesthesia with respect to the incidence of postoperative delirium following hip surgery [139, 140]. However, some researchers have suggested clinicians to consider combining epidural and general anesthesia rather than general anesthesia alone in patients undergoing major thoracic and abdominal surgeries to reduce the risk of postoperative delirium [141].

### Comparing analgesic techniques

Thoracic epidural block (TEB) and paravertebral block (PVB) are commonly used in clinical practice. These two regional blockade techniques showed equal effectiveness in controlling postoperative acute pain in patients undergoing thoracotomy surgery. However, PVB was superior in reducing delirium, as demonstrated in a study where spinal anesthesia with bupivacaine was performed on patients who received knee arthroplasty and supplemented with propofol or dexmedetomidine for sedation [142]. This was further verified in another study on elderly patients undergoing elective surgery for video-assisted thoracoscopic lobectomy (VATS), who received general anesthesia maintained with inhaled sevoflurane and intravenously infused remifentanil. In this study, thoracic paravertebral block (TPVB) with 0.2% ropivacaine showed superior effects on pain control and inhibition of perioperative stress and inflammatory response, and a significantly lower incidence of delirium, and a higher rate of QoR on POD7, as compared to intravenous analgesia with 2 µg/kg sufentanil [143]. Notably, TPVB has also minimized opioid consumption, which is beneficial because the use of opioids is highly pertinent to the development of delirium in a dose-dependent manner [144, 145].

### Non-pharmacological strategy

Electroacupuncture (EA) is an alternative acupuncture technique where applies an electric current to inserted needles at different acupoints [146]. As an adjuvant therapy to conventional anesthetics, EA treatment has its application in postoperative pain control. Recently, it has been proposed as a therapeutic strategy for POCD due to its capability of relieving cognitive dysfunction while preserving hippocampal neurons via inhibiting activation of NLRP3 inflammasome [147].

### Traditional Chinese medicines (TCMs)

In recent years, increasing number of natural extracts have been tested for their potential application as preventive and therapeutic approaches for PND.

*Baicalin* is extracted from the dried rhizome of *Astragalus membranaceus* and is the primary pharmacological component of astragalus, with anti-viral, anti-inflammatory, anti-apoptotic and anti-oxidative functions. Administering Baicalin at various doses to aged rats demonstrated alleviated cognitive dysfunction after splenectomy via anti-inflammatory mechanisms and pathways involving N-methyl-D-aspartate (NMDA) receptor 2B regulatory subunit, which mediates LTP and synaptic plasticity [148].

*Gastrodin *(*GAS*) is a natural compound with multiple functions of sedation, analgesia, anti-epilepsy, anti-depression and memory improvement. When pre-operatively and post-optatively administering GAS to aged mice subjected to laparotomy, it improved learning and memory by suppressing microglial activation and phosphorylation of GSK-3β and Tau, suggesting its neuroprotective role in the prevention and treatment of POCD [149].

*Cistanche* (Rou Cong Rong in Chinese) has been used as a tonic in China for many years. It showed anti-apoptosis, anti-oxidation, anti-ageing, anti-fatigue, immunomodulatory anti-inflammatory and neuroprotection in the CNS [150]. Intraperitoneal injection of Cistanche to aged rats attenuated sevoflurane-induced hippocampus-dependent memory impairments by activating PPARɣ signaling and suppressing microglial activation [10].

*Curcumin* is an active compound derived from *Curcuma Longa* with antioxidant effects. It ameliorated the cognitive dysfunction in aged mice undergoing abdominal surgery and neutralized cholinergic dysfunction induced by surgery [151].

*Berberine* is an isoquinoline alkaloid purified from Chinese herbs with anti-inflammatory effects and readily crosses the BBB. Berberine rescued surgery-induced cognitive impairment and inhibited hippocampal release of proinflammatory cytokines in the in-vivo model, and suppressed LPS-stimulated production of proinflammatory cytokines in the in-vitro model [152].

*The root extract of valeriana officinalis* has anti-inflammatory properties [153], stimulatory effects on serotonin (5-HT) and acetylcholine (ACh) receptors [154] and therapeutic effects on sleep disturbance [155]. Patients scheduled for elective on-pump CABG surgery exhibited a better cognitive performance at early post-surgery stage after treated with CPB with the root extract, indicating its potential therapeutic value for POCD [156].

*Glycyrrhizin* is the major component of *Glycyrrhiza glabra* with a good bioavailability and BBB penetration, making it possible to treat neurological diseases through oral administration [157]. Additionally, it binds directly to HMGB1 to inhibit its chemoattractant and mitogenic activities [158]. The intraperitoneal treatment of glycyrrhizin attenuated isoflurane-induced cognitive deficits in neonatal rats [159], whereas oral pretreatment with glycyrrhizin showed a prevention of POCD by inhibiting HMGB1-induced neuroinflammation and AD-related pathology in hippocampus of aged mice subjected to splenectomy surgery [160].

*Honokiol* ([2-(4-hydroxy-3-prop-2-enyl-phenyl)-4-prop-2-enyl-phenol]) is a bioactive compound extracted from species of *Magnolia*, used as an activator of Sirtuin3 (SIRT3) with multiple properties including anti-tumor, anti-arrhythmic, anti-thrombolytic, anti-inflammatory, anti-angiogenesis, anxiolytic, anti-oxidative activities in both in vivo and in vitro studies [161, 162]. Honokiol attenuated surgery-induced memory loss via antioxidant and anti-inflammatory pathways in hippocampus of a POCD mouse model [163].

### Other types of drugs

#### Immunomodulatory medications

Thalidomide can easily cross the BBB after acute systemic administration, and dramatically reduces the expression and synthesis of multiple proinflammatory cytokines in the peripheral and central nervous systems [164]. Intraperitoneal administration of a single-dose of thalidomide immediately after laparotomy has significantly accelerated recovery from acute postoperative pain and reversed deterioration in spatial memory function at POD 14 on aged POCD rats through its long-term regulation of NMDA receptors in hippocampus [165]. Some immunosuppressive drugs have also been tested. For instance, intracisternal administration of the IL-6 antagonist tocilizumab at the time of surgery attenuated the inflammatory response and improved cognitive outcome in rats [45].

### Antioxidants

Chronic inflammation has been previously revealed to develop oxidative stress that contributes to cognitive dysfunction [166]. Previous studies have shown that surgical trauma was able to induce oxidative stress [15] and contribute to development of POCD [167]. In addition, the BBB has been shown to be interrupted by peripheral oxidative stress [168], and the BBB breakdown has been associated with cognitive impairment [167]. Furthermore, animal model of tibial fracture showed increased protein oxidative damage in prefrontal cortex and hippocampus, along with impaired memory [33]. All these studies indicated a potential role of oxidative stress in development of cognitive dysfunction in POCD and a potential beneficial effect of antioxidants. The utilization of antioxidants in management of POCD remains to be largely unexplored, but studies have started to emerge. Edaravone, a known antioxidant, has been demonstrated to antagonize POCD both in patients [169] and in surgery- or LPS-treated rats [170], possibly due to its antioxidant and anti-inflammatory effects, as well as its ability to maintain activation of the Akt/mTOR signaling pathway. Further studies are warranted on the effects of antioxidants on progression of POCD.

### Anti-psychotic medication

Quetiapine is an atypical antipsychotic medication with activity at dopaminergic (D2), histaminergic (H1), serotoninergic (5-HT1A, 5-HT2A), and noradrenergic (alpha 1) receptor sites, and it has been utilized to manage the symptoms of delirium. Quetiapine showed preserved reversal learning on LPS-challenged rat model, which may be associated with the preservation of downstream effects related to noradrenergic-mediated cortisol suppression in the treatment of acute delirium [171]. Topiramate (TPM) was a sulfamate-substituted monosaccharide drug often used to treat epilepsy and migraine. This drug showed neuroprotective effects in POCD rats and beneficial reactions in primary hippocampal microglia of these rats with apoptotic properties [172].

### Iron-chelator

Iron homeostasis is critical in maintaining CNS function including oxygen transport, neurotransmission, myelination and neuronal metabolism [173]. However, iron accumulation occurs in ageing brain, leading to oxidative stress and neurotoxicity, as shown in neurodegenerative disorders [174]. Treatment with a potent iron chelator Deferoxamine (DFO) significantly reduced microglia activation and proinflammatory cytokine release and alleviated subsequent hippocampal-dependent memory deficit in mice after surgery, possibly through p38 MAPK signaling pathway [175].

### Stem-cell-based therapies

Mesenchymal stem cells (MSC) are often used in regeneration and tissue repair [176], and MSC-conditioned medium (MSC-CM) has anti-inflammatory and antioxidative properties [177]. Intravenous injection of MSC-CM ameliorates PND in mice subjected to left liver lobectomy, with reduced level of IL-1β, IL-6, TNF-ɑ and malondialdehyde and increased level of BDNF in the brain tissue [178].

### GABAergic system

Anesthesia- and surgery-induced hippocampal neuroinflammation can disrupt the GABAergic system, which increased the expression of surface ɑ5-subunit-containing subtype of GABAARs (ɑ5GABAARs) via the p38 MAPK signaling pathway, and eventually led to hippocampus-dependent memory dysfunction. In a PND rodent model, blocking ɑ5GABAARs by L655708 or blocking p38 MAPK by SB203580 alleviated laparotomy-induced cognitive deficits with a reduction of p-P38 and surface ɑ5GABAARs [179].

### Statins

Atorvastatin-treated PND mice showed improved cognitive function (particularly fear response and spatial memory) with attenuated neuroinflammation associated with increased PPARɣ expression [180] and phosphorylation and inactivation of neuronal GSK3β [181] in hippocampus. Ulinastatin (UTI) is a multivalent Kunitz-type serine protease inhibitor, which is also called the urinary trypsin inhibitor. When given to rats before or after isoflurane exposure, UTI attenuated isoflurane-impaired learning capacity and neuronal apoptosis, whereas pre-treatment seemed to be more effective [182]. Perioperative multiple UTI infusions have been tested in elderly patients undergoing elective spinal surgery, showing a significant reduction of POCD incidence with lower serum levels of LPS, IL-6, CRP and MMP-9, as well as shortened peak value duration [183].

### Gas compound

Methane, the most abundant organic gas compound on earth and the most common bacterial metabolic product with redox regulation and attenuation of mitochondrial dysfunction, could penetrate the BBB [184] or the blood-spinal cord barrier [185], making it a promising therapy for CNS disorders. Methane has been found as a novel agent for POCD via its anti-inflammatory properties [186]. Hydrogen sulfide (H_2_S), which is traditionally a toxic gas, protects neurons against oxidative stress [187] and attenuates LPS-induced cognitive impairment by reducing the overproduction of proinflammatory mediators [188]. Recently, S-propargyl-cysteine, a novel hydrogen sulfide-modulated agent, have been demonstrated to attenuate LPS- or Aβ-induced spatial learning and memory impairment [189]. When administering sodium hydrosulfide (NaHS) as hydrogen sulfide (H_2_S) donor to a rodent model prior to surgery, it significantly attenuated surgery-induced memory impairment and expression of proinflammatory cytokines both in serum and in hippocampus [190].

### CB2R agonist

Expression of cannabinoid receptor type 2 (CB2R) under neuroinflammatory conditions has been observed downregulated in the brain, particularly in microglia [191], thus, activation of CB2R is believed to dampen the production of inflammatory mediators and to facilitate the production of prosurvival factors [192]. In adult mice subjected to intramedullary fixation surgery for tibial fracture under isoflurane anesthesia, postoperative treatment with CB2R agonist (JWH133) attenuated surgery-induced memory loss, whereas CB2R antagonist (AM630) aggravated surgery-induced memory loss, paralleled with a decreased or increased expression of proinflammatory factors in hippocampus and prefrontal cortex [193].

## Future directions

Since there is currently no standard preventive and therapeutic strategies for PND, more preclinical and clinical data are required. Targeting neuroinflammation seems to be a promising direction. Based on the reported studies, we should bear in mind some key points when designing future studies. Firstly, drug selection. From surgical and anesthesic challenge to postoperative neurocognitive disorders, a series of complicated pathophysiological events are involved. Neuroinflammation is a well-documented key player for the pathogenesis of PND, however, specific targets should be properly and carefully selected to reach a long-term clinical value. This is the main aim of this review. Secondly, brain regions. To date, many studies have focused on surgery- and anesthesia-induced neuroinflammation and neurotoxicity in hippocampus, an important brain region for learning and memory. However, the effects of anesthesia, surgery and ageing on the brain did show regional differences in previous studies [194, 195]. Specifically, it has been previously suggested that prefrontal, frontal, parietal, temporal, occipital cortex, hippocampus, insula, cingulate, thalamus and cerebellum were all involved in POCD [194]. In addition, the hippocampus may be related to cognitive processes such as declarative memory, while the dorsolateral prefrontal cortex may be related to processes such as working memory [195]. Together, these studies indicate that multiple brain regions may be involved in post-surgery neurocognitive dysfunctions, which required further investigations. Thirdly, study models. The characteristics of models (e.g., age, timepoint of neurocognitive changes) are needed to be considered when selecting PND models for the study. For instance, in an abdominal surgery model, microgliosis and cognitive deficits were visible on POD7 and disappeared in 2 or 3 weeks after operation in young rats, but continued to POD14 [196] or even 6 weeks after surgery [197] in aged rats. More constant alterations are often seen in cardiac surgery models compared to non-cardiac surgery models. In a CPB model, cognitive impairments can persist for at least 6 months after surgery in association with constant neuroinflammation and reduced adult neurogenesis during the same period [198]. The genetic studies with inbred rodents have provided insights into mechanisms worth investigating as playing potential roles in human PND, however, these studies are just models. Moreover, older adults are much, much more complex than genetically inbred young mice, and these mouse models (unless they are performed in outbred older animals with comorbid health conditions) overemphasized the role of procedural factors and underemphasized the role of patient factors and comorbidities in human PNDs. Fourthly, marker selection. To monitor neuroinflammation, pro-inflammatory markers, including IL-1β, IL-6 and TNF-ɑ, are often monitored. It is noteworthy that the time points of the individual cytokine activation may vary significantly. For instance, circulating IL-6 levels remained elevated up to 72 h after surgery, whereas IL-1β level only appeared elevated on POD14 [12]. Finally, concomitant medications. The use of opioids is highly pertinent to the development of POD in a dose-dependent manner, so total consumption of opioids such as fentanyl should be compared when designing clinical trials.

## Conclusions

Neuroinflammation has profound pathophysiological mechanisms and effects on the development and progression of PND. A variety of drug candidates have been tested in both preclinical experiments and clinical trials, aiming to find out novel preventive and therapeutic strategies for PND or to add new indications to approved drugs. Both safety issues and drug efficacy should be further investigated in large-scaled clinical trials, particularly for their long-term effects. Therefore, more efforts should be made to provide a clearer picture for the correlation between neuroinflammation and pathogenesis of PND, and how specific drug candidates work.

## Data Availability

The authors declare that the relevant data are included in the article.
